# Dynamic Initiation and Propagation of Multiple Cracks in Brittle Materials

**DOI:** 10.3390/ma6083241

**Published:** 2013-07-31

**Authors:** Jie Li, Qiaoping Huang, Xiaodan Ren

**Affiliations:** 1 Department of Building Engineering, Tongji University, 1239 Siping Road, Shanghai 200092, China; E-Mail: rxdtj@tongji.edu.cn; 2 Tongji Architectural Design (Group) Co., Ltd., 1239 Siping Road, Shanghai 200092, China; E-Mail: huangqp81@yahoo.com

**Keywords:** brittle materials, dynamic fracture, fragmentation, mechanical properties, strain rate effect, dynamic damage evolution

## Abstract

Brittle materials such as rock and ceramic usually exhibit apparent increases of strength and toughness when subjected to dynamic loading. The reasons for this phenomenon are not yet well understood, although a number of hypotheses have been proposed. Based on dynamic fracture mechanics, the present work offers an alternate insight into the dynamic behaviors of brittle materials. Firstly, a single crack subjected to stress wave excitations is investigated to obtain the dynamic crack-tip stress field and the dynamic stress intensity factor. Second, based on the analysis of dynamic stress intensity factor, the fracture initiation sizes and crack size distribution under different loading rates are obtained, and the power law with the exponent of −2/3 is derived to describe the fracture initiation size. Third, with the help of the energy balance concept, the dynamic increase of material strength is directly derived based on the proposed multiple crack evolving criterion. Finally, the model prediction is compared with the dynamic impact experiments, and the model results agree well with the experimentally measured dynamic increasing factor (DIF).

## 1. Introduction

Dynamic fracture of brittle materials is of special interest in the physics and materials community [1,2,3,4]. Under rapid loading, brittle materials tend to break into pieces. The higher the loading rate is, the smaller the sizes of fragments are [5,6,7,8]. Much work has been done to investigate the physical mechanism of this phenomenon, including the dynamic fracture experiments [9,10,11,12,13], numerical simulations [14,15,16] and theoretical developments [17,18,19,20,21].

So far, the physical explanation of rate sensitivity of brittle materials is still an open field with many issues remain ambiguous. Mott [16] was the first to study the dynamic fragmentation in an analytical way. In Mott’s model, the concept of material randomness and stress relief were introduced. The fragmentation of brittle materials was formed by the competition between the dynamic stress loading and stress relief. This idea was further extended by Hild and his co-workers to develop an alternative probabilistic damage model [22]. In their model, local cracks are randomly nucleated as a time dependent process. Then each nucleated crack propagates at a certain velocity and relieves a neighboring region whereas further fracture in the region is prohibited. Although this statistical model considers the physical mechanism of crack propagation, it ignores the dynamics of fracture initiation.

On the other hand, the question of stress wave propagation in an elastic solid with a single isolated crack has been well discussed by Sih and Loeber [23]. In the work of Chen [24], the dynamic response of crack to various types of Heaviside loadings has been developed. Based on this method, Kipp and his co-workers [25] explained the dynamic strength increase of oil shale under high strain rate. While these dynamic fracture based methods reveal some important features of crack initiation, further investigations on crack initiation size and diffusion of cracking under dynamic impact from the view point of material physics are lacking, and a proper physically based model is needed to explain and describe these phenomena.

In the present work, based on the methods proposed by Sih and Kipp [23,25], an analytical relation between minimum crack initiation size and loading rate is proposed by studying crack tip stress wave propagation. Furthermore, the phenomenon of multiple crack propagation under dynamic loading is well explained and described. The equation to describe the dynamic increase of material strength is then developed.

The paper is organized as follows. In Section 2, the dynamic response of a single crack embedded in an infinite homogeneous elastic body under stress wave loading is investigated to obtain the fracture initiation sizes and the crack size distribution under different loading rates. Based on this knowledge, and combined with the concept of energy balance in facture, a multiple crack propagation dynamic fracture model is proposed, and the dynamic increasing factor for brittle materials can be derived easily. In Section 3, comparison between model prediction and experimental results is provided, and numerical results show that the present model is capable of describing the phenomenon of multiple fragmentations and strain rate effect physically, and a few conclusions are drawn in the last section.

## 2. Mechanical Modeling

### 2.1. Fracture Initiation and Multiple Cracking

**Figure 1:**
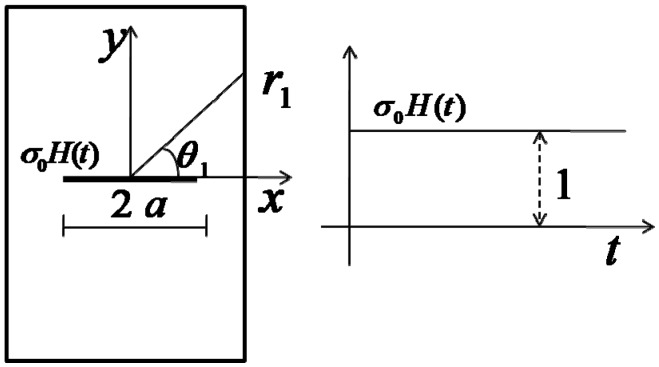
Crack geometry and loading.

For the sake of simplicity, the brittle material is idealized as a homogenized elastic body with a single embedded crack. The same treatment can be also found in many work including Sih and Loeber [23]. So, consider here an infinite isotropic elastic body containing a through plane crack with the length of 2a subjected to impact tensile loading normal to the crack surfaces. The transient response of the crack-tip will be obtained by using the solution of the Heaviside normal traction applying on the upper and lower crack surfaces (Figure 1).

**Figure 2:**
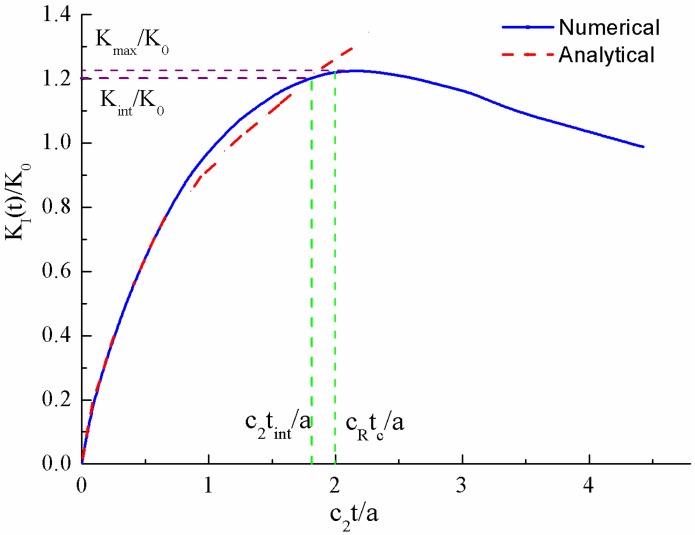
Dynamic stress intensity factor.

As shown in Sih and Loeber [23], the Laplace transform is introduced to solve the wave equation. The specific response at the crack tip of the dynamic stress intensity factor may then be formulated in terms of a standard Fredholm integral equation of the second kind. In Figure 2, the dynamic stress intensity factor, normalized with respect to the corresponding static value of stress intensity factor *K*_0_ is plotted as a function of *c*_2_*t*/*a* (the blue line), where *c*_2_ is shear wave speed. For brittle materials like oil shale, the shear wave speed is about 1773.6 m/s and the ratio of shear wave speed to dilatational wave speed is about 0.612. It is noted that the dynamic intensity factor reaches its maximum rapidly and then decreases in amplitude oscillating about the static intensity factor (Figure 2).

Before the Rayleigh waves interact to each other the transient response of crack tip dynamic stress intensity factor could be approximated by the semi-infinite crack solution [24], which is characterized by the square root of the time:

(1) $$K_{I}(t)\sim \sqrt{t}$$

Specifically, for the mode I fracture, the dynamic stress intensity factor could be derived in the form:

(2) $$K_{I}(a,t)=\frac{2\alpha \sigma_{0}\sqrt{a}}{\sqrt{\pi }}\sqrt{c_{2}t/a}$$

The Heaviside loading response could then be readily employed as Green’s function for the general dynamic loading [18]. Specifically, for the constant strain rate loading, the corresponding dynamic stress intensity factor at crack tip (red dash line in Figure 2) could be written as:

(3) $$K_{I}(t)=\alpha \frac{2\sqrt{a}}{\sqrt{\pi }}{\dot{\sigma }}_{0}\int_{0}^{t}\sqrt{c_{2}\tau /a}d\tau =2\alpha {\dot{\sigma }}_{0}\sqrt{\frac{c_{2}}{\pi }}t^{3/2}$$

where, denotes the geometry coefficient obtained from the early portion of the inversion numerical curves in Figure 2.

**Figure 3:**
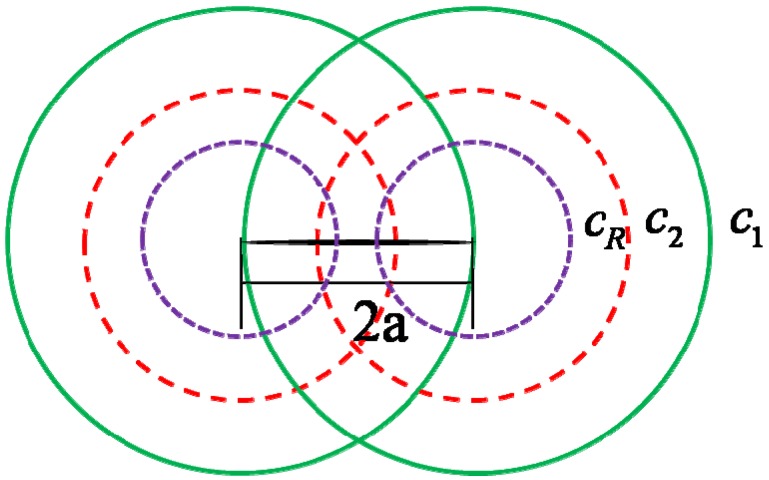
Wave fronts emanating from crack tip.

Before we go further to the dynamic fracture, we should analyze the dynamic time history of stress field distribution around the crack-tip. When the Heaviside normal traction loading is applied to the crack surfaces, both of the crack-tips generate two outgoing cylindrical waves, e.g., the dilatational wave and the shear wave. At the same time, a surface wave that travels along the crack surfaces is also formed. Here, *c*_1_, *c*_2_ and *c*_R_ denote the dilatational wave speed, shear wave speed and the Rayleigh surface wave speed, respectively. The dynamic stress wave propagation pattern is shown in Figure 3. However, it should be noted that the analytical solution of the dynamic stress intensity factor shown in Equation (3) is identical to the solution of the semi-infinite crack under impact before the dilatational wave reaches the other tip of the crack [20,23,25]. Upon the arrival of the dilatational wave front the dynamic stress intensity factor tends to divert slightly from the semi-infinite solution, because of the wave interaction. Then an intersection between the numerical solution and the analytical solution could be observed in Figure 2. We denote the normalized dynamic stress intensity factor at the intersection as $K_{\mathrm{int}}$ and also denote the corresponding dimensionless time as $c_{2}t_{\mathrm{int}}/a$. According to the conclusions drew by Freund [20], the dynamic stress intensity factor decreases when the Rayleigh wave reaches the other tip of the crack. Thus we define the maximum dynamic stress intensity factor as $K_{\max}$ while the corresponding dimensionless time could be well approximated by $c_{R}t_{c}/a$ [20]. The maximum value of dynamic stress intensity factor $K_{\max}$ is critical because it is required by the fracture criterion under dynamic loading. However, it is hardly possible to solve it analytically. Thus, in the present paper, we approximate $K_{\max}$ by calculating the analytical expression of Equation (3) at the dimensionless time point $c_{R}t_{c}/a$. As can be observed from Figure 2, our approximation agrees not too bad with the numerically solved $K_{\max}$. Similar treatment can be found in the work of Kipp and coworkers [25].

It is observed that the dynamic stress intensity factor calculated by Equation (3) may be large enough and exceed the critical stress intensity factor ${\text{K}}_{\text{IC}}$, while the crack growth initiates. So, the critical time $t_{s}$ at which $K_{\text{I}}(t)$ reaches ${\text{K}}_{\text{IC}}$ can be expressed as:

(4) $$t_{s}={\left(\frac{3\sqrt{\pi }{\text{K}}_{\text{IC}}}{4\alpha \sqrt{c_{2}}{\dot{\sigma }}_{0}}\right)}^{\frac{2}{3}}$$

By using the linear elastic stress-strain relation, the dynamic strength is obtained as:

(5) $$\sigma_{c}={\left(\frac{9\pi {\text{E}}_{0}{\text{K}}_{\text{IC}}^{2}}{16\alpha^{2}c_{2}}{\dot{\varepsilon }}_{0}\right)}^{\frac{1}{3}}$$

where, ${\text{E}}_{0}$ is the Yong’s modulus, and ${\dot{\varepsilon }}_{0}$ is the loading rate. In order to investigate the influence of crack geometry on the dynamic strength, a sensitivity analysis of Equation (5) has been performed. For common brittle materials, it is revealed that the dynamic strength is insensitive to geometry coefficient for different crack shapes, and this is in agreement with Kipp [25]. One could also see that, for a given strain rate, the dynamic strength is independent of the initial crack size.

In order to determine the minimum critical size of the crack evolving at a given loading rate, the critical time expressed in Equation (4) should be reexamined. One must note that, Equation (1) only holds when the time period is shorter than the period of time $t_{c}$. According to the aforementioned analysis, the dynamic stress intensity factor $K_{I}(t)$ reaches its maximum at $t_{c}$. Thus, we conclude that the $K_{I}(t)$ of the evolving crack should reach ${\text{K}}_{\text{IC}}$ before the critical time $t_{c}$. We have:

(6) $$t_{s}\leq t_{c}\Rightarrow {\left(\frac{3\sqrt{\pi }{\text{K}}_{\text{IC}}}{4\alpha \sqrt{c_{2}}{\dot{\sigma }}_{0}}\right)}^{\frac{2}{3}}\leq \frac{2a}{c_{R}}$$

So, for a given loading rate ${\dot{\sigma }}_{0}=E_{0}{\dot{\varepsilon }}_{0}$, the minimum critical crack size is obtained as follows:

(7) $$a_{c}\geq 0.5\tilde{k}{\left(\frac{3\sqrt{\pi }{\text{K}}_{\text{IC}}c_{2}}{4\alpha E_{0}}\right)}^{\frac{2}{3}}{\dot{\varepsilon }}_{0}^{-\frac{2}{3}}$$

It is noted that, the minimum critical crack size commits to the power law with the exponent parameter to be −2/3. The same exponent parameter is also obtained from the experiment results of Gilvarry and Bergstrom [26], Hayakawa’s numerical simulation [27], and Grady’s theoretical conclusion [7], in which the power law distribution was employed by experience to describe the fragmentation size or mass.

**Figure 4:**
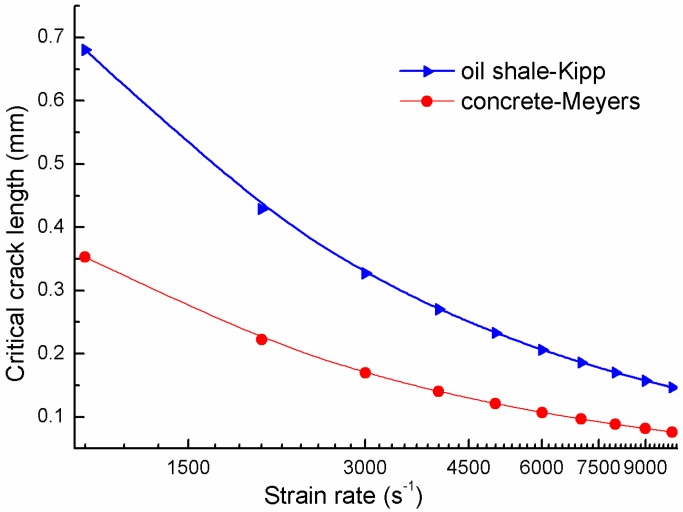
Critical crack length *vs.* loading rates.

Figure 4 shows the relation between the minimum critical crack size and the strain rate in the range 10^3^ s^−1^~10^4^ s^−1^ for concrete and oil shale. The material parameters are taken from Kipp [25] and Meyers [8]. It is seen from the curves that the minimum critical size decreases as the loading rate increases. This means that the relatively small preexisting cracks may participate to growth under higher loading rate.

**Figure 5:**
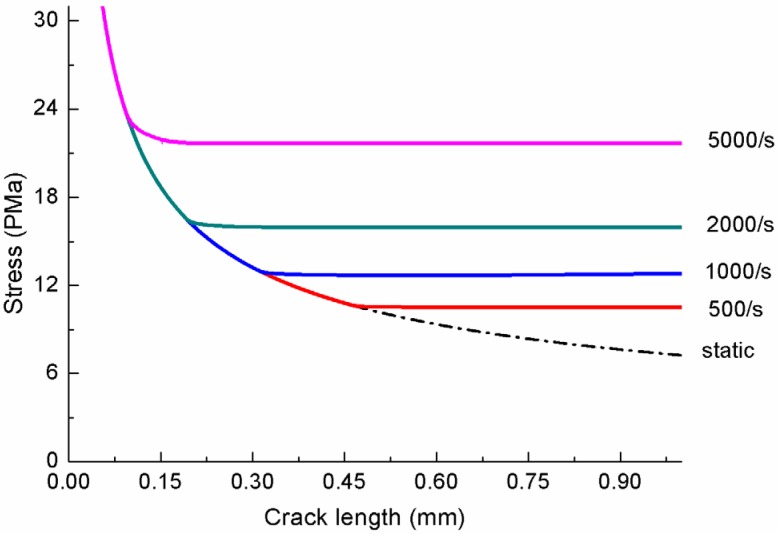
Fracture stress *vs.* crack size at constant strain-rate loading.

The fracture stress *vs.* crack size curves are plotted in Figure 5 for several representative strain rates. The static stress-crack length relationship is also plotted in Figure 5 (${\dot{\varepsilon }}_{0}$ = 0) for reference. It is observed that, as the strain rate increases, the points of departure between the dynamic and static solutions move toward smaller crack radii, and the higher fracture stress levels are achieved (higher dynamic strength). One can also see that, for each strain rate (except static loading), there is an intermediate crack length for which the fracture stress reaches the minimum, *i.e.*, a preferential crack size. In other words, when a solid with an array of cracks is loaded statically, the largest crack will dominate the response of the solid, limiting the maximum load that can be applied. If a preferred orientation of the largest flaws exists, the material will also show orientation dependence to the fracture stress. In the dynamic case, however, the largest crack no longer dominates; rather, cracks with a wide range of sizes are clearly activated simultaneously. So, the failure occurs by fracturing the solid through multiple crack growth. Even with some preferred flaw orientations, the dynamic fracture stress tends to be orientation independent [25]. The material parameters used to calculate the curves in Figure 5 are: ${\text{E}}_{0}$ = 37.0 GPa, *K*_IC_ = 0.67 MPa $\sqrt{\text{m}}$, $c_{2}$ = 1773.6m/s, $\tilde{k}$ = 0.911.

**Figure 6:**
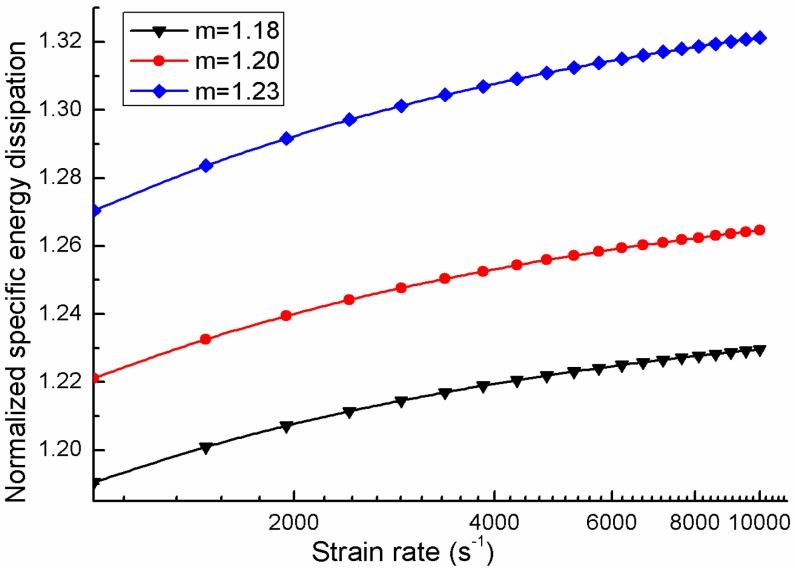
Logarithmic normalized specific energy dissipation with loading rates.

Research has indicated that the propagation pattern for cracks is fractal and the growth of such cracks is multifractal [28,29,30,31,32]. Xie [33] have showed that the density function of the distribution for crack length is fractal, and can be described by:

(8) $$p\left(\frac{a_{i}}{a}\right)=\lambda_{0}{\left(\frac{a}{a_{i}}\right)}^{1+m/2}$$

where, *λ*_0_ and *m* are material parameters, 2*a* is the largest crack length that is considered, and 2*a_i_* are the crack length of smaller cracks. Oddershede [34] have shown that, the *m* of gypsum is around 1.2. Here in order to investigate the influence of parameter *m* on the energy dissipation during the process of multiple cracking, *m* is taken the same value as suggested by Oddershede. Thus we have *m* equals to the values of 1.18, 1.20 and 1.23. The normalized specific energy dissipation is:

(9) $$\bar{S}=\frac{S}{\gamma }=\sum_{a=a_{c}}^{\infty }4a_{i}$$

where, γ is the free surface energy density of the material. Substituting Equations (7) and (8) into Equation (9), one can get the normalized specific energy dissipation of multiple cracking materials. The relation between the normalized specific energy dissipation for gypsum and loading rates for different m are shown in Figure 6.

It is found that, the higher the loading rate is, the more energy is dissipated by cracking. This is due to the fact that higher loading rate can activate smaller preexist crack so that more energy is dissipated. This in accordance with the physical fact that smaller fragments always account for a larger numbers of fractions in the material.

Hogan *et al.* [35] carried out the dynamic fracture experiments for the natural polyphase ceramic to investigate the dynamic fracture character of brittle materials. In their experiments, specimens were subjected to high strain rate. It was observed that the higher the loading rate yields smaller fragments and smaller dominant length scale in their probability distributions. This means that many cracks with different sizes are propagating simultaneously and smaller per-existing crack are activated to propagate at higher loading velocity. Meanwhile, the measured total generated fracture surface area increases with the increase of loading rate, indicating that the energy required to create the new surface at higher loading rate is generally greater than that under lower loading rate. Thus, the energy dissipated in the fracture process is greater than that of static fracture. Similarly, a series of dynamic fracture experiments of PMMA (polymethylmethacrylate) carried out by Scheibert *et al.* [36] also indicated that fracture energy is increasing with the increase of loading rate. In addition, numerical simulations of dynamic fracture also reveal the same phenomena [37].

### 2.2. Multiple Dynamic Fracture model

In Section 2.1, we have shown that many pre-existing cracks of different sizes tend to propagate simultaneously when subjected to dynamic loading. With this knowledge, similar to the classical Griffith static fracture theory for the plane stress problem, consider a system that contains an array of micro-cracks whose propagation pattern for cracks is fractal. Ignoring the interaction potential between neighboring cracks, the energy balance equation of solid with multiple-crack propagation under dynamic loading is:

(10) $$\frac{d\tilde{U}}{da}=\frac{d}{da}[\frac{\pi a^{2}\sigma^{2}}{E}+\sum_{i=1}^{N}\frac{\pi a_{i}^{2}\sigma^{2}}{E}],(a_{i}\leq a)$$

where, 2a is the crack length of the largest crack, $2a_{i}$ are the crack length of smaller cracks, $\tilde{U}$ is the potential strain energy of the system, σ is the stress and is E the Young’s modulus of the considered material.

According, the surface energy per unit thickness of the crack system is written as:

(11) $$\tilde{S}=4\gamma (a+\overset{N}{\underset{i=1}{\sum }}a_{i}=4a\gamma (1+\overset{N}{\underset{i=1}{\sum }}\frac{a_{i}}{a})$$

As the propagation pattern for cracks is fractal as described by Equation (8), the follow relation must be committed:

(12) $$\frac{da_{i}}{a_{i}}=\frac{da}{a}$$

Substituting Equation (12) into Equation (11), then, for a small increase of crack length, the increase of the surface energy is:

(13) $$\frac{d\tilde{S}}{da}=4\gamma (1+\overset{N}{\underset{i=1}{\sum }}\frac{a_{i}}{a})$$

In particular, when there is only one crack, the energy balance equation takes the classical form:

(14) $$\frac{dU}{da}=\frac{2\pi a\sigma^{2}}{E}$$

Substituting Equations (12) and (14) into Equation (10), then, for the multiple dynamic cracking, the equilibrium for a unit growth of main crack can be written as:

(15) $$\frac{d\tilde{U}}{da}=\frac{dU}{da}[1+\sum_{i=1}^{N}{\left(\frac{a_{i}}{a}\right)}^{2}]\Rightarrow \frac{dU}{da}=\frac{4(1+\sum_{i=1}^{N}\frac{a_{i}}{a})}{1+\sum_{i=1}^{N}{\left(\frac{a_{i}}{a}\right)}^{2}}\gamma =\tilde{\gamma }$$

where, $\tilde{\gamma }$ is the equivalent surface energy density of the material and U is the potential strain energy. Recalling Equation (8), and with the assumption that, the crack size is a continuous variable, the equivalent surface energy density can be written as:

(16) $$\tilde{\gamma }=\frac{1+\int_{x_{c}}^{1}\lambda_{0}{\left(1/x\right)}^{1+\frac{m}{2}}xdx}{1+\int_{x_{c}}^{1}\lambda_{0}{\left(1/x\right)}^{1+\frac{m}{2}}x^{2}dx}\gamma$$

where, $x=a_{i}/a$ is the normalized variable, and $x_{c}=a_{c}/a$; According to static fracture mechanics, the relationship between the largest crack length a and the material strength is:

(17) $$a=\frac{1}{\pi }{\left(\frac{K_{IC}}{\sigma_{t}^{s}}\right)}^{2}$$

where, $\sigma_{t}^{s}$ is the static fracture strength of material.

So, the dynamic strength increase of material reads:

(18) $$\frac{\sigma_{\;t}^{d}}{\sigma_{\;t}^{s}}\propto \sqrt{\frac{\tilde{\gamma }}{\gamma }}\propto \sqrt{\frac{1+\int_{x_{c}}^{1}\lambda_{0}{\left(1/x\right)}^{1+\frac{m}{2}}xdx}{1+\int_{x_{c}}^{1}\lambda_{0}{\left(1/x\right)}^{1+\frac{m}{2}}x^{2}dx}}=k_{dif}$$

where, $\sigma_{t}^{d}$ is the dynamic fracture strength of material.

## 3. Numerical Implementation and Results

**Figure 7:**
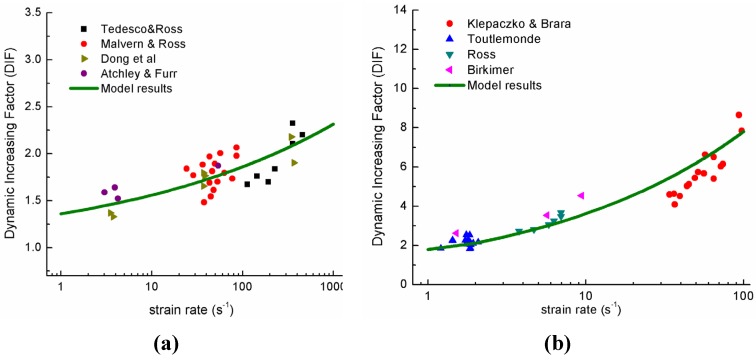
(**a**) Dynamic increasing factors (DIFs) under compression; and (**b**) DIFs under tension [39,40,41,42,43].

As we all know, the compression failure of brittle materials is due to the shear failure of materials. By replacing the mode I fracture (tensile fracture) with mode III fracture (shear fracture), the dynamic shear fracture can obtained. The experimental observed dynamic increasing factors (DIF) for compression and tension are collected to validate the proposed model. Figure 7a shows the model results DIF in dynamic compression and Figure 7b shows the model results DIF in dynamic tension. The used material parameters of compression and tension considered in the present study is listed in Table 1 [38].

**Table 1:** Material parameters of concrete under dynamic compression and tension.

E(GPa)	*υ*	*ρ* (Kg/m^3^)	*K*_IC_ (MPa $\sqrt{m}$)	*c*_2_ (km/s)
30.0	0.2	2500	0.63	1.74
$\tilde{k}$	*λ_t_*_0_	*m_t_*	*λ_c_*_0_	*m_c_*
0.911	4500	22.0	3000	4.3

where, $\lambda_{t0}$ and $\lambda_{c0}$ denote the $\lambda_{0}$ in tension and compression, $m_{t}$ and $m_{c}$ denote the m in tension and compression respectively.

## 4. Conclusions

In the framework of dynamic fracture mechanics, by studying the response of a single crack under stress wave impact, the phenomenon of multiple cracking of brittle materials under dynamic loading is modeled. Experimental validation of the proposed model is also given. It is observed that the model results agree well with experimental data, thus demonstrating the following capabilities: (1) The minimum fracture initiation size and its distribution under different loading rates are obtained by analyzing the pattern of crack tip wave propagation; (2) The power law distribution of the fracture initiation size is derived with the exponent to be −2/3, which is a well accepted result for the dynamic fragmentation distribution; (3) The conclusion that, under high loading rate, cracks of different sizes in brittle materials tend to propagate simultaneously can be obtained, based on which, the physical phenomenon of multiple fragmentation for brittle materials under high loading rate can be well explained; (4) By introducing the fractal distribution of crack length and energy equilibrium of the dynamic fracture system, the dynamic increase of stress intensity factor and material strength is derived. The calculated dynamic increasing factor represents the salient tendencies of the experimental data.
